# Factors affecting knowledge regarding unmet need on fertile aged women in Indonesia: evaluation of 2012 and 2017 IDHS

**DOI:** 10.1186/s12978-022-01338-5

**Published:** 2022-01-29

**Authors:** Achmad Kemal Harzif, Mila Maidarti, Fransisca Novi Handayaning, Azizah Fitriayu Andyra

**Affiliations:** 1 Department of Obstetrics and Gynecology, Cipto Mangunkusumo National Referral Hospital, Faculty of Medicine, University of Indonesia, Jakarta, Indonesia; 2 Indonesian Reproductive Medicine Research and Training Center (INA- REPROMED) Faculty of Medicine Universitas Indonesia, Dr. Cipto Mangunkusumo Hospital, Jakarta, Indonesia

**Keywords:** Family planning, Contraception, Unmet need

## Abstract

**Background:**

The Family Planning (FP) Program is a national method of controlling population growth rates while improving maternal and child health. Indonesia, as one of the largest countries, has abysmally low contraceptive coverage. One of its main issues is unmet contraceptive needs. This study aims to determine the factors that influence women's unmet need of childbearing age (WCA) in Indonesia.

**Methods:**

We performed an unpaired comparative analytic study with a cross-sectional method was conducted on secondary data obtained from 2012 to 2017 Indonesia Demographic and Health Survey (IDHS). The subjects in this study were all women of childbearing age (15–49 years). Subjects with incomplete data were excluded from the study. Unmet need was defined as WCA who did not use contraception but decline to have more children or wanted to delay their pregnancies. Chi-square analysis was performed on categorical data and Mann–Whitney U analysis on numerical data.

**Result:**

A total of 45,607 WCA in the 2012 IDHS data and 29,627 WCA in the 2017 IDHS data were included in the study. In the 2012 IDHS data, factors influencing unmet needs were age (p = 0.023) and parity (p < 0.0001). In the 2017 IDHS data, factors influencing unmet needs were the residential area (p = 0.003), level of education (p = 0.008), level of spouse’s education (p < 0.0001), employment status (p = 0.03), possession of electricity (p = 0.001), and possession of television (p = 0.01).

**Conclusion:**

Factors affecting unmet needs are age, parity, residential area, level of education, level of spouse’s education, employment status, possession of television, and possession of electricity**.** There were no recurring factors on 2012 and 2017 IDHS data.

## Plain language summary

The Family Planning (FP) Program is a national method of controlling population growth rates while improving maternal and child health. Indonesia, as one of the largest countries, has abysmally low contraceptive coverage. One of its main issues is unmet contraceptive needs. This study aims to determine the factors that influence women's unmet need of childbearing age (WCA) in Indonesia.

An unpaired comparative analytic study with a cross-sectional method was conducted on secondary data obtained from 2012 and 2017 Indonesia Demographic and Health Survey (IDHS). The subjects in this study were all women of childbearing age (15–49 years). Subjects with incomplete data were excluded from the study. Unmet need was defined as WCA who did not use contraception but decline to have more children or wanted to delay their pregnancies. Chi-square analysis was performed on categorical data and Mann–Whitney U analysis on numerical data. A total of 45,607 WCA in the 2012 IDHS data and 29,627 WCA in the 2017 IDHS data were included in the study. In the 2012 IDHS data, factors influencing unmet needs were age and parity. In the 2017 IDHS data, factors influencing unmet needs were the residential area, level of education, level of spouse’s education, employment status, possession of electricity, and possession of television.

In conclusion, factors affecting unmet needs are age, parity, residential area, level of education, level of spouse's education, employment status, possession of television, and possession of electricity. There were no recurring factors on 2012 and 2017 IDHS data.

## Background

It is estimated that the global population would reach 7 billion people, And with increasing life expectancy every year, it is predicted to continue to grow and reach 8 billion in 2023 [1, 2].

Over the past 20 years, the usage of contraceptives in developing countries has decreased the number of maternal mortality simply by reducing unplanned pregnancies [3]. This action directly decreases the number of illegal abortions and high-risk pregnancies. Nevertheless, these successes are far from perfect. Previous research states that as many as 30% of maternal deaths can be further reduced by meeting unmet needs for contraception [3]. In addition, contraception may also increase perinatal outcomes by increasing the interval between pregnancies [3, 4].

Methods and forms of pregnancy planning differ widely, starting from traditional techniques, such as periodic abstinence, disrupted intercourse, and methods from myths and beliefs to modern techniques that have been studied for their efficacy. The intrauterine device (IUD), condoms, hormonal (pills), implants, and birth control injections are some well-known pregnancy planning approaches in the culture. There are also modern procedures, such as vasectomy and tubectomy, which are not commonly known or even feared by the public [5].

Based on data in 2017, traditional contraceptive methods were used by 4.6% of women of childbearing age (WCA) in Indonesia, while modern methods were used by 41.4% of WCA. The most widely used modern methods are injection (20.9%), pill (8.7%), IUD (3.5%), and implant (3.4%). Other methods, such as the lactational amenorrhea method (LAM) and male sterilization, were only used by 0.1% of all WCA [6, 7].

Unmet need is one of the persistent problems found in every country related to the provision of contraceptive services. Unmet need is defined as WCA who decline to have more children or delay pregnancies but do not use contraception [6, 8].

The level of unmet need varies from country to country, with a higher percentage in developing countries such as Uganda, Haiti, and Ghana [9]. Based on 2014 data, it was found that the amount of unmet need in Indonesia ranged from 10 to 11%, more or less the same as other Asian countries [9].

Previous studies have shown that several interventions may be utilized to increase contraceptive use rates. However, unmet need is one of the most prevalent problems to be addressed. Currently, there were only a few studies regarding unmet contraceptive needs in Indonesia. This study aims to determine the factors influencing unmet needs in Indonesia.

## Methods

An analytic observational study with a cross-sectional method was done using re-analysis of 2012 and 2017 Indonesia Demographic and Health Survey (IDHS) raw data. The study population was WCA, whose data was recorded on IDHS. Patients with incomplete records were excluded from this study. 45,607 subjects were recorded on 2012 IDHS, while 29,267 subjects were recorded on 2017 IDHS.

Risk factors analyzed in this study were age, parity, history of sexually transmitted disease, residential area, level of education, level of spouse’s education, employment status, socioeconomic status, possession of electricity, radio, television and cellphone, smoking status, and the willingness of discussing puberty with daughter. Unmet need is defined as WCA who did not use any form of modern contraception but decided to delay or prevent birth.

Baseline characteristics were then analyzed and compared. Bivariate analysis between subjects’ characteristics and contraceptive knowledge was done. Multivariable analysis was done to determine factors associated with contraceptive knowledge and unmet need. Ethical clearance was issued from the health research and ethical committee in Faculty of Medicine, University of Indonesia.

## Results

Using the raw data of Indonesian Demographic and Health Survey (IDHS), 45,607 respondents from 2012 IDHS data and 29,267 respondents from 2017 IDHS data were analyzed. Table 1 (2012 IDHS) and Table 2 (2017 IDHS) investigated the relationship between characteristics of subjects and unmet needs.

**Table 1:** Relationship between characteristics and Unmet need in 2012 IDHS

Characteristics	Unmet need		*p*	OR (CI 95%)
	Yes	No		
Age	35 (15–49)	31 (15–49)	< 0.001	
Parity	2 (0–13)	1 (0–14)	0.001	
Sexually transmitted disease history				
Yes	1 (2.6%)	37 (97.4%)	0.270	0.42 (0.06–3.09)
No	2.717 (6.0%)	42.559 (94.0%)		
Residential area				
City	1.402 (5.9%)	22.404 (94.1%)	0.322	0.96 (0.89–1.04)
Rural	1.332 (6.1%)	20.469 (93.9%)		
Education				
Uneducated	110 (7.3%)	1.390 (92.7%)	< 0.001	
Primary	1100 (7.3%)	14.025 (92.7%)		
Junior high	1194 (5.1%)	22.236 (94.9%)		
Senior high	331 (6.0%)	5.222 (94.0%)		
Education (years)	6 (0–6)	5 (0–6)	< 0.001	
Spouse’s education				
Uneducated	69 (7.1%)	909 (92.9%)	< 0.001	
Primary	1.010 (7.4%)	12.664 (92.6%)		
Junior high	1.287 (7.4%)	16.045 (92.6%)		
Senior high	342 (9.7%)	3.191 (90.3%)		
Employment status				
Working	1.558 (6.2%)	23.701 (93.8%)	0.084	1.07 (0.99–1.16)
Not working	1.176 (5.8%)	19.164 (94.2%)		
Socioeconomic status				
First quintile	621 (8.0%)	7.146 (92.0%)	< 0.001	
Second quintile	513 (5.8%)	8.272 (94.2%)		
Third quintile	483 (5.2%)	8.760 (94.8%)		
Fourth quintile	535 (5.5%)	9.208 (94.5%)		
Fifth quintile	583 (5.8%)	9.488 (94.2%)		
Possession of electricity				
Yes	2.527 (5.8%)	40.796 (94.2%)	< 0.001	0.51 (0.43–0.61)
No	152 (10.8%)	1.260 (89.2%)		
Possession of radio				
Yes	823 (5.2%)	15.091 (94.8%)	< 0.001	0.79 (0.73–0.86)
No	1.852 (6.4%)	26.937 (93.6%)		
Possession of television				
Yes	2.275 (5.8%)	37.289 (94.2%)	< 0.001	0.72 (0.64–0.80)
No	406 (7.8%)	4.776 (92.2%)		
Smoking				
Yes	102 (9.5%)	969 (90.5%)	< 0.001	0.16 (0.14–0.20)
No	2.629 (5.9%)	41.897 (94.1%)		
Puberty discussion				
Yes	348 (8.3%)	3.859 (91.7%)	0.559	1.05 (0.90–1.22)
No	370 (7.9%)	4.294 (92.1%)

**Table 2:** Relationship between characteristics and Unmet need in 2017 IDHS

Characteristics	Unmet need		*p*	OR (CI 95%)
	Yes	No		
Age	36 (15–49)	32 (15–49)	< 0.001	
Parity	2 (0–13)	1 (0–13)	0.001	
Sexually transmitted disease history				
Yes	6 (6.5%)	86 (93.5%)	0.097	1.17 (0.51–2.69)
No	2.771 (5.6%)	46.590 (94.4%)		
Residential area				
City	1.434 (5.6%)	24.109 (94.4%)	0.858	0.99 (0.92–1.07)
Rural	1.361 (5.7%)	22.723 (94.3%)		
Education				
Uneducated	53 (6.4%)	770 (93.6%)	< 0.001	
Primary	888 (6.5%)	12.675 (93.5%)		
Junior high	1.501 (5.5%)	25.998 (94.0%)		
Senior high	351 (4.5%)	7.390 (95.5%)		
Education (years)	6 (0–6)	5 (0–12)	< 0.001	
Spouse’s education				
Uneducated	59 (10.5%)	504 (89.5%)	< 0.001	
Primary	850 (7.1%)	11.083 (92.9%)		
Junior high	1.511 (8.0%)	17.296 (92.0%)		
Senior high	330 (7.7%)	3.934 (92.3%)		
Employment status				
Working	1.492 (5.6%)	24.955 (94.4%)	0.932	1.00 (0.93–1.08)
Not working	1.302 (5.6%)	21.850 (94.4%)		
Socioeconomic status				
First quintile	523 (6.2%)	7.941 (93.8%)	0.097	
Second quintile	549 (5.8%)	8958 (94.2%)		
Third quintile	550 (5.5%)	9.539 (94.5%)		
Fourth quintile	563 (5.3%)	10.020 (94.7%)		
Fifth quintile	609 (5.5%)	10.375 (94.5%)		
Possession of electricity				
Yes	2.621 (5.6%)	44.488 (94.4%)	0.013	0.75 (0.60–0.94)
No	85 (7.3%)	1.084 (92.7%)		
Possession of radio				
Yes	563 (5.0%)	10.665 (95.0%)	0.002	0.86 (0.78–0.95)
No	2.142 (5.8%)	34.885 (94.2%)		
Possession of television				
Yes	2.461 (5.5%)	42.089 (94.5%)	0.010	0.83 (0.73–0.96)
No	244 (6.5%)	3.483 (93.5%)		
Access to internet				
Yes	1.095 (4.6%)	22.813 (95.4%)	< 0.001	0.68 (0.62–0.73)
No	1.657 (6.6%)	23.337 (93.4%)		
Possession of cellphone				
Yes	2.121 (5.5%)	36.769 (94.5%)	0.002	0.86 (0.79–0.95)
No	665 (6.2%)	10.014 (93.8%)		

Subsequently, a multivariable analysis was done between characteristics and unmet needs. The result could be seen in Table 3 (2012 IDHS) and Table 4 (2017 IDHS).

**Table 3:** Multivariable analysis of Unmet need 2012 IDHS

Characteristics	*p*	OR	CI 95%
Age < 20 years	0.023	1.30	1.04–1.62
Parity: nulliparity	< 0.0001	8.96	7.17–11.20

**Table 4:** Multivariable analysis of Unmet need 2017 IDHS

Characteristics	*p*	OR	CI 95%
Residential area: city	0.003	0.89	0.82–0.96
Education: uneducated	0.008	0.68	0.51–0.90
Spouse’s education: uneducated	< 0.0001	14.58	11.76–18.09
Employment status: working	0.030	0.92	0.85–0.99
Possession of electricity: no	0.001	1.30	1.12–1.52
Possession of television: no	0.010	0.82	0.71–0.95

## Discussion

In this study, it is clear that numerous factors were affecting unmet needs in WCA. The family planning program is a program that has succeeded in increasing contraceptive use by as much as 60% in couples worldwide [10]. It is estimated that there are 225 million women in the world whose contraceptive needs are still not being met each year. The situation is unfortunate, considering that contraception in an unmet need population can further prevent 36 million abortions, 70,000 maternal deaths, and 52 million unwanted pregnancies [10].

Age is one of the factors that determine the use of contraception. Previous research focusing on women aged 15–24 has shown that contraceptive knowledge and use among younger women tend to be lower, especially when combined with lower education and rural areas [11, 12]. Previous studies have also shown that this is related to more significant concern on younger women and would translate into lower contraceptive coverage in the younger age category [13].

Education, spouse’s education, and possession of various facilities (electricity, radio, television, cellphone, and internet) are linked to the availability of information flows that reach the WCA. Previous research in Bangladesh and Ghana has shown that education is a very influential factor in the use of contraception because women with higher levels of education tend to have a better understanding of the benefits and risks of using contraception [14, 15]. Better education would also lead to higher levels of contraceptive use [14, 16, 17].

Afterward, it was also known that factors associated with unmet needs are age, parity, residential area, level of education, level of spouse’s education, employment status, possession of television, and possession of electricity. The number of unmet needs is directly related to the number of unplanned and unwanted pregnancies. Previous research has shown a 16-fold chance of developing an unwanted pregnancy in women with unmet needs [17].

Age, parity, education, spouse's education, and access to information would influence the incidence of unmet needs in Indonesia. Previous research conducted in Indonesia in 2015 also showed similar results that age and parity would determine the incidence of unmet need in WCA [18]. Therefore, further education is needed, not only about family planning and contraceptive programs but also the ideal number of children for couples [19].

One of the considerations affecting the decision on contraceptive use is the characteristics of the spouse. As one of the countries with strong patriarchal values, WCA in Indonesia has difficulties ranging from accessing school and sexual education to not having the right to determine the number of children deemed appropriate [10]. In this report, women with lower spouse’s education are more likely to be identified as an unmet need. Previous research has shown that women in developing countries appear to be rejected by their spouses, who desire more offspring. They also have many obstacles and must struggle harder in order to have access to contraception [10, 20].

In conclusion, factors affecting unmet needs range from intrinsic characteristics such as age and parity to spouse’s characteristics such as education and socioeconomic status. There were no recurring risk factors. However, the risk factors multiplied in the later years. Comprehensive education and contraceptive provision would be beneficial to improve the rate of contraceptive use in Indonesia.

## Conclusions

Factors affecting unmet needs are age, parity, residential area, level of education, level of spouse’s education, employment status, possession of television, and possession of electricity. No recurring factors were affecting unmet need on 2012 and 2017 IDHS data.

## Data Availability

All data generated or analysed during this study are included in this published article.

## Abbreviations

FB: Family Planning
WCA: Women of childbearing age
IDHS: Indonesia demographic and health survey
IUD: Intrauterine device
LAM: Lactational amenorrhea method
